# Key drivers and priorities of consumer decisions for refurbished electronics: A mix-method approach

**DOI:** 10.1016/j.heliyon.2024.e40977

**Published:** 2024-12-06

**Authors:** Fatemeh Barkhi, Sadra Ahmadi, Sajad Shokouhyar, Raffaele Filieri, Masoud Ramezaninia

**Affiliations:** a Department of Information and Industrial Management, Faculty of Management and Accounting, Shahid Beheshti University, G.C., P.O. Box 1983963113, Tehran, Iran; b Cyberspace Research Institute, Shahid Beheshti University, G.C., P.O. Box 1983963113, Tehran, Iran; c Department of Supply Chain and Operations Management, Australian Institute of Business, Adelaide, Australia; d Department of Marketing, Audencia Business School, 8 Rte de la Jonelière, 44300, Nantes, France

**Keywords:** Refurbished product, Consumer attitudes, Social media, Data mining, Consumer purchasing decisions

## Abstract

Product refurbishment enhances waste minimization and environmental sustainability. However, the sale of these products relies on consumer attitudes, influenced by various factors. This study adopts a novel mixed-methods approach, utilizing structural and network analysis based on over 60,000 comments and tweets from X.com (formerly Twitter), complemented by insights from expert interviews, to analyze factors affecting consumers viewpoints when making decisions about purchasing refurbished electronics. The research identifies and prioritizes 17 key influencing factors, highlighting the interplay between large-scale consumer sentiment and expert opinions. A customer-centric purchase decision model is presented, showing that consumer priorities when purchasing refurbished products typically revolve around retailer reputation, brand, pricing, warranty, and product quality. These findings offer valuable guidance for producers on how to target higher-priority consumer concerns, ultimately helping to expand their market and boost purchasing intention.

## 1 Introduction

Refurbishing and reintroducing products to the market provides consumers with environmental and financial benefits [1] while enabling producers to offer modern items at a more competitive price than new products. Electronic waste (e-waste) is the fastest-growing solid waste stream in the world, and each year, over 50 million tons of e-waste are produced, which causes severe damage to human health [2].Valuable components and materials can be repurposed through refurbishment, restoring used products through collection, isolation, repair, and replacement [3]. By reducing energy and material consumption, refurbishment not only contributes to environmental preservation but also results in cost savings for consumers [4]. Producers strive to create a market for refurbished products and require an in-depth analysis of consumer behavior in choosing between refurbished and new items [5].

Refurbishing restores returned products to like-new condition [6], involving the renewal of used electrical and electronic equipment through inspection, repair, and cleaning to extend their lifespan. The role of refurbishment is crucial in managing a closed-loop supply chain (CLSC) as it effectively decreases the transfer of waste and cuts down on associated costs [7]. For example, laptop refurbishing repairs or replaces damaged parts, which is popular in the electronics sector due to its non-destructive and energy-saving advantages [8].

According to Agostini, Bigliardi [9] and Mahmud [10] consumer attitudes are key in enhancing consumers' intention to purchase refurbished products. However, little is known about the key factor driving consumers’ refurbished products adoption, especially in the electrical and electronic equipment industry. Studies like Harms and Linton [11] and Mugge, Jockin [12] that used survey methods identified various factors influencing consumer intention to buy refurbished products. For instance, low price, perceived value and perceived risk [13], disgust caused by physical contact with a previous owner [14], quality and warranty, the presence of an eco-friendly certification [11], product functionalities was considered noticeably affecting consumers mindsets. Additionally, seller reputation and attitudes towards refurbished products [9], customer behavior, customer value, green awareness, government policies, technological advancements, and reverse logistics [15] had also significant impact on consumers decision making.

However, little research has investigated behavior, which is increasingly considered an important variable in understanding the intention-behavior gap in most studies on green product purchases [16]. Recent studies using large-scale data analysis in service sectors have revealed important patterns in online consumer behavior [17,18]. Online consumer comments which is especially useful for understanding environmentally conscious behaviors, provide a rich source of authentic feedback, offering valuable insights into actual customer actions [19].

This study proposes a novel methodological approach to understanding consumer attitudes toward refurbished products by combining large-scale data analysis from social media with expert interviews. By analyzing over 60,000 comments and tweets and leveraging network analysis and structural methods, we aim to uncover the key factors driving purchasing decisions and develop a decision model that will help manufacturers better meet market demand.

Social media data is adopted because consumers increasingly use and rely on social media to gather opinions about consumer goods [20], especially in the realm of experiential goods like electronics. X has become essential for understanding consumer behaviors and motivations [21,22], with 14.5 million daily active users in the U.S. during the second quarter of 2022 [23], marking a significant rise from 2021. It allows users to share opinions in text format, distinguishing it from platforms requiring photo or video uploads. Also, it provides valuable unstructured data for analyzing environmental shifts, market dynamics, societal trends, and consumer attitudes, helping organizations develop better strategies [24].

To summarize, this research aims to make the following contributions.

1 Analyzing consumer attitudes toward electronic refurbished products posted on social media, which include a wide assortment of positive and negative opinions.
2 Identify the factors based on consumers' posts on social media that affect consumer decisions to buy refurbished products.
3 Identifying interrelationships between influential factors affecting consumers' decision to purchase refurbished products and prioritizing them using structural methods (ISM) and network analysis (Gephi)
4 Creation of a purchase decision model founded on ranked factors and examining the feasibility of purchasing or cancelling the purchase of refurbished products.

## 2 Literature review

In recent times, the refurbishment of products has been receiving gradually more attention in academic discourse, emerging as a critical endeavor for reducing the environmental footprint of waste in sectors spanning construction and electronics [25]. Refurbished products, sold at reduced prices after collection, lessen environmental and social impacts [26].

Consumer mindsets and behaviors are at the core of successful engagement with circular consumption systems. Gomes, Moreira [27] reveals that environmental awareness and price sensitivity are critical factors influencing consumer attitudes toward refurbished products. However, these behaviors are highly context-dependent, requiring businesses to be mindful of the specific factors driving consumer decisions in their target markets. Wallner, Haslbeck [28], investigate factors such as perceived risk and financial incentives, showing that consumers are more hesitant to purchase refurbished products when concerns about hygiene or prior ownership (territorial contamination) are present, especially in personal electronics like earbuds. This suggests that addressing perceived risk and offering value-driven incentives are crucial for boosting the adoption of refurbished products.

Zaremohzzabieh, Ismail [29] explores the role of consumer attitudes, environmental knowledge, and awareness in shaping green purchase intentions, including the decision to buy refurbished products. The study emphasizes the importance of promoting positive attitudes toward refurbished products by focusing on their environmental benefits. Price sensitivity and perceived value are significant determinants of consumers' willingness to purchase refurbished products. Harms and Linton [11], demonstrate that eco-certification significantly increases consumer willingness to pay for refurbished items. This is especially relevant for environmentally conscious consumers who prioritize sustainable choices.

de Vicente Bittar [30] argues that while environmental consciousness influences some consumer decisions, factors such as price and brand equity are more critical in driving sales. This finding suggests that while promoting environmental benefits is important, businesses must focus on competitive pricing and brand reputation to appeal to a broader consumer base. This aligns with the insights from Esmaeilian, Saminathan [3], which explores probabilistic selling as an effective marketing strategy for refurbished products. By offering refurbished items at lower prices with uncertain product features (e.g., model or condition), companies can attract cost-sensitive consumers. Mugge, Jockin [12], identify specific consumer incentives, such as improved battery life, software updates, and performance guarantees, as key motivators for purchasing refurbished smartphones. Different consumer groups respond to different incentives, highlighting the importance of tailored marketing strategies to effectively target diverse consumer segments.

Consumer knowledge about the refurbishment process plays a crucial role in shaping purchasing behavior. Wang, Zhu [31] shows that providing detailed information about the refurbishment process increases consumer willingness to switch from new to remanufactured products. Transparency regarding the product history and refurbishment procedures helps reduce perceived risk and builds consumer confidence, although price and performance often remain the primary drivers of consumer decisions.

Cultural and regional factors also influence consumer attitudes toward refurbished products. Seifian, Shokouhyar [32] explores how consumer behavior towards refurbished mobile phones differs between the USA and India. It finds that Americans prioritize product characteristics, while Indians place more importance on seller-related aspects. Chun, Matsumoto [33] examines consumer behavior in Japan and Indonesia, finding that price sensitivity and perceived risk are key factors. The study highlights that Japanese consumers show less brand loyalty toward refurbished smartphones compared to Indonesian consumers, who have a stronger preference for OEM-branded refurbished products. These cultural differences indicate the need for region-specific marketing strategies to promote refurbished products in diverse markets. Geopolitical factors, such as supply chain disruptions caused by the Russo-Ukrainian war, can also impact consumer perceptions. Obadă, Dabija [34], discuss how geopolitical events influence consumer attitudes toward recycled and refurbished electronics, particularly by affecting product availability and pricing. This highlights the importance of external factors in shaping consumer behavior and attitudes toward sustainable products.

The sustainability of refurbished products is a growing concern for consumers in both developed and developing countries. Ghorbanloo and Shokouhyar [35] reveals that while consumers in developing regions prioritize economic benefits, those in developed countries are more concerned with environmental sustainability. This finding underscores the importance of adapting marketing strategies to the specific concerns of consumers in different regions. Niu and Xie [36] examine how competition and the remanufacturer's quality reputation influence incentives for certifying refurbished products, finding alignment in competitive markets and when quality image disparity is substantial.

The importance of social media and online platforms in shaping consumer behavior is explored in Mariani and Nambisan [37]. This study emphasizes the role of big data and online reviews in understanding consumer preferences and feedback regarding refurbished products. Companies can use these insights to refine their marketing strategies and address consumer concerns about quality and value. Social media engagement also plays a crucial role in influencing consumer satisfaction. Uzir, Jerin [38] find that consumers who engage with brands on social media are more likely to express satisfaction with refurbished products, especially when they perceive value for money. This suggests that active social media engagement can enhance consumer trust and satisfaction with refurbished goods.

Many previous studies relied mainly on questionnaires and interviews to gather consumer opinions (Table 1), leading to limited sample sizes that fail to capture diverse customer groups. In contrast, analyzing virtual social media platforms offers the benefit of gathering perspectives across nationalities and languages without the limitations of sample size or time [39].

**Table 1:** Summary of the relevant literatures.

Article	Product	Methodology	Key findings
Wallner, Haslbeck [28]	Speakers and earbuds	Survey	Perceived risk, product category, and territorial contamination
Chun, Matsumoto [33]	Smartphones	Online survey	Perceived risk, Price consciousness, Environmental Consciousness
Nasiri and Shokouhyar [40]	Smartphones	1 Online survey 2 Reviews from e-commerce websites	Function, Warranty, Appearance, Price, Battery health, Accessory, Packaging, Environment
Zwicker, van Harreveld [41]	Mobile phone	Online survey	Psychological factors (joy and green product interest, product status and uncertainty)
Mugge, Jockin [12]	Smartphones	Online survey	Battery, performance, screen, camera, internal storage, innovative features
Agostini, Bigliardi [9]	Smartphone	Online survey	Seller reputation and distribution, perceived value and risk
Wallner, Magnier [42]	Headphones	Choice-based conjoint analysis	State of the product, warranty, battery, price,
Esmaeilian, Saminathan [3]	Cellphone	Survey analysis	Price, Battery, Weight, Operating System, Color, Item Dimension, Screen Size GSM, Condition, Customer Rating
Radi and Shokouhyar [43]	Mobile phone	1 Social Media Analysis 2 Content analysis, Descriptive Analysis and Sentiment analysis	Environment, social responsibility, economic
Alyahya, Agag [44]	Refurbished products	Fuzzy set qualitative comparative analysis (FsQCA)	Moral obligation, moral accountability, moral outrage " perceived severity, perceived venerability, egoistic value, and attitude, rewards, perceived cost, and altruistic value

Moreover, prior research often overlooked the interrelationships between factors and the priorities that influence consumer decision-making. To bridge these gaps, this study employs a mixed-method approach to provide deeper insights into consumer attitudes toward refurbished products and develop a theoretical framework for decision-making. Existing studies (Table 1) have identified key factors influencing consumer intentions to purchase refurbished electrical and electronic products, forming a basis for further exploration.

## 3 Methodology

**Fig. 1:**
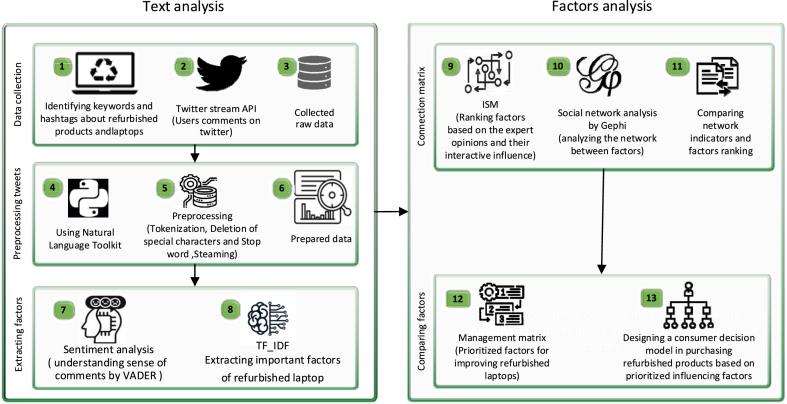
Steps of the proposed research method.

Data was gathered utilizing X.com's Application Programming Interface (API) and underwent a two-stage analysis procedure (Fig. 1). During the initial phase, Sentiment Analysis and Term Frequency-Inverse Document Frequency (TF-IDF) were employed to identify factors influencing consumer attitudes. In the following stage, interpretive structural modeling and network analysis were conducted using Gephi to explore the intricate relationships among the factors identified in the initial step. Lastly, the outcomes obtained from the interpretive structural modeling and Gephi network analysis were employed to prioritize and compare factors using a management matrix.

### 3.1 Textual analysis

#### 3.1.1 Phase 1: gathering data

The team obtained a X.com developer's license, employing Python (NLTK) for data collection. Python's extensive libraries facilitate natural language processing and data analysis [45]. Specific keywords from Table 1 were used in Python code to selectively gather relevant user comments, ensuring targeted data collection after authorized access to tweets.

Table 2 outlines the main factors and associated minor related topics that influence consumers' purchasing decisions when selecting electronic devices. The primary factors are essential attributes that buyers consider, while each main factor is further detailed with minor related topics that represent specific characteristics or features contributing to the overall value of that factor.

**Table 2:** Factors and keywords related to refurbished products and laptop.

Main Factor	Minor Related Topics
Price	Purchase
Memory size	Capacity, internal, Storage, Space, Memory
Screen resolution	LCD, Display, Widescreen, Liquid Retina, Multi-Touch, Contrast Ratio, TrueDepth, Brightness
Number of USB ports	Ports, USB
Quality	Quality, Functionality, History, Standard Grade, Status, Feature, State, Usage, Lag
Battery	Power, Battery Life, Rechargeable, Lithium-Ion Battery, Fast-Charge, Qi Wireless Chargers, USB-C Power Adapters, Battery Performance, Energy-Saving, Battery Health
Special features	Touch pad, Backlit Keyboard, Fingerprint Reader, Memory Card Slot, Numeric Keypad
Wireless communication	Bluetooth, Wi-Fi
Mother board	Mother board
Operating system	Update, Control center, Auto-Brightness, General, Accessibility, Animations, Background Activity, Location Services, Lock Screen, Home Screen, Airplane Mode, Dark Mode, Augmented Reality, Reminders, Settings
Weight	Weight
Screen display size	Width, Depth, Inch
CPU model	CPU, Core i5, Core i7, Intel, AMD
Retailers' reputation	reputation, retailer, familiar, recognized
Warranty	Warranty, AfterSale, Quarantee, Quaranty
Hard disk type	HDD hard, SSD hard
Brand	Manufacturer, Famous, Known

#### 3.1.2 Phase 2: the preprocessing of tweets and analysis of sentiment

Data preprocessing is crucial in data mining to enhance clarity and reduce ambiguity, involving tasks like removing retweets, tokenization, lowercasing, eliminating stop words, deleting coordinates, stemming, and excluding non-emotional digits and characters [46]. A compilation of example tweets from users is presented in Table 3.

**Table 3:** Examples of tweets in their raw, unprocessed form.

No.	Tweets
**1**	"Not sure I will ever buy a new one again" … sounds like someone has joined the Refurbished Revolution!
**2**	This was my second laptop from them. I choose “good”. In reality it was perfect
**3**	It works perfectly and delivered quickly !! My daughter loves it. Not sure I will ever buy a new one again !?
**4**	Ahh here we go again!! Good job Acer with you worst service can't even get my repaired laptop. If this is your service refund me the amount of the laptop and keep the trash with yourself!!
**5**	I have only/one month left #refurbishedlaptop and display not working properly.
**6**	I know there's a company here in Ireland that sell refurbished laptops called Green IT for less than you'd normally pay. There might be a UK equivalent?!!!
**7**	Hey it's alright I'm planning to get a refurbished apple laptop from eBay? You do what you can until you can afford to get a laptop that is up to date. Apple to me is the best hence tons of 2010 on sale on eBay!!!

During this phase, the Natural Language Toolkit (NLTK) is used among Python libraries to execute procedures for analyzing human language data, offering essential tools for text processing like classification, markup, and semantic analysis [43]. Data acquired via the API in JSON (JavaScript Object Notation) format is converted to Comma-Separated Values (CSV) for analysis (Table 4).

**Table 4:** Cells in the CSV file containing output.

Field of data	Characterization
**ID**	Unique identifier for tweets
**Created_at**	Date and time of the tweet
**Text**	Content of the tweet
**Sentiment**	Sentiment expressed in the tweet
**Polarity**	Polarity of the sentiment
**Lang**	Language used in the tweet
**Retweet_count**	Number of retweets
**Hashtag**	All hashtags extracted from the tweet
**Place**	User's location

Tweet sentiment analysis aims for accurate classification into sentiment categories using lexicon-based methods and machine learning algorithms. Among lexicon-based approaches like SentiWordNet [47], and Valence Aware Dictionary and sEntiment Reasoner (VADER) [48], VADER stands out for labeling words as positive, negative, or neutral based on their context within a sentence. It utilizes a Compound score, ranging from −1 (highly negative) to +1 (highly positive), derived by summing individual word ratings. This score provides a standardized measure of sentiment, facilitating classification into predefined positive, neutral, or negative categories. The threshold values commonly used are as follows.

1. If the compound score is greater than or equal to 0.05, classify as Positive
2. If the compound score is between −0.05 and 0.05, classify as Neutral
3. If the compound score is less than or equal to −0.05, classify as Negative

#### 3.1.3 Phase 3: factor extraction

In earlier studies, various machine learning techniques such as TF-IDF (Term Frequency-Inverse Document Frequency), Word2vec, and Bag of Words were used for data classification. TF-IDF extracts features by assessing the frequency and significance of keywords in a text compared to their presence across a corpus, assigning weights to document words to highlight the most relevant ones in sentiment analysis [49].

The steps to calculate the weight of words and repeat them in this manner are as follows (Eq. (1),(2):

(1) $$TF=\frac{1+\log\left(k\mathrm{e}y\;word\;count\right)}{\log\left(word\;count\right)}$$

**TF (Term Frequency)** = The number of times a word has been used per docking.

(2) $$IDF=\log\left(1+\frac{total\;docum\mathrm{e}nt}{document\;with\;keyword}\right)$$

**Inverse Document Frequency (IDF)** = Reverse the number of documents in which a word is used.

**Fig. 2:**
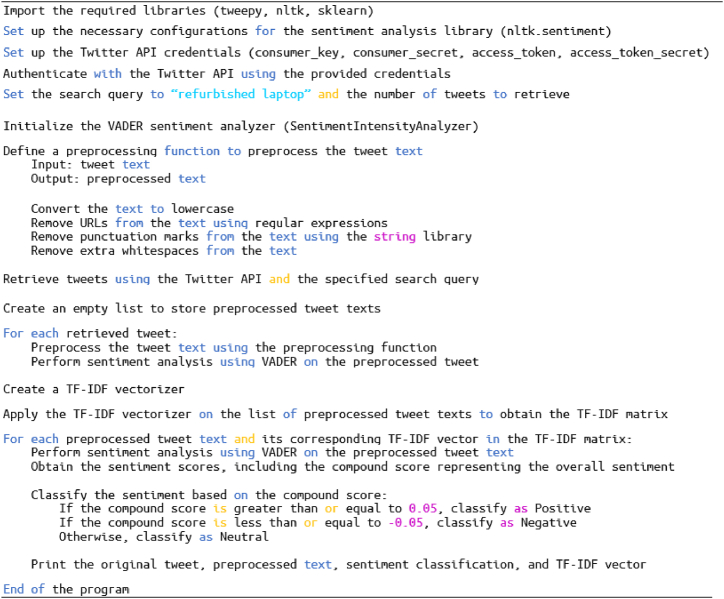
Pseudocode tailored to gather tweets about refurbished products and laptops.

A total of seventeen crucial factors influencing consumer perception and attitude towards refurbished products and laptops were derived. The pseudocode for each step is elucidated as follows (Fig. 2).

### 3.2 Factor analysis

#### 3.2.1 Phase 4: interpretive structural modeling

The Interpretive Structural Modeling (ISM) method analyzes interconnections among factors influencing consumer attitudes toward refurbished products. As a Multiple Criteria Decision-Making (MCDM) technique, ISM identifies influential factors and their interrelationships for decision-making in management and operational research [50]. In our study, ISM was employed to identify key factors and reveal their contextual relationships.

The implementation of the ISM tool involved a series of steps as follows.

• Step 1: Considering seventeen factors initially.
• Step 2: Establishing detailed relationships with a SSIM using V, A, X, and O relationship types.
• Step 3: Converting the SSIM into a binary form (0 and 1) to derive an initial reachability matrix.
• Step 4: Conducting a transitivity check for consistency (following the transitivity rule that necessitates the relationship between factors A, B, and C if A is related to B, and B is related to C.)
• Step 5: Segmenting the final reachability matrix into levels to categorize factors.
• Step 6: Establishing a hierarchical structure based on these levels to understand relationships among factors.

#### 3.2.2 Phase 5: MICMAC analysis

The MICMAC analysis validates indirect relationships identified through interpretative structural modeling (ISM), focusing on interconnections between factors. By graphically illustrating factor influence, MICMAC ensures precise conclusions and highlights significant and minor driving factors within complex systems. This combined approach has been widely used across diverse domains, including barriers to green supply chain management [51] and others. The MICMAC analysis classifies factors into four distinct categories:

***Autonomous Factors***: Displaying limited driving power (DRP) and dependence power (DNP), indicating weak connections with the system.

***Dependent Factors***: Possessing modest driving power but strong dependence power, relying heavily on other factors within the system.

***Linkage Factors***: Demonstrating robust driving and dependence power, serving as vital connectors linking various elements of the system.

***Driver Factors***: Also known as independent factors, exhibiting considerable driving power but weak dependence power, exerting substantial influence on other factors within the system.

#### 3.2.3 Phase 6: network analysis of factors (Gephi)

The study initially identified factors influencing consumer attitudes toward refurbished products and laptops. Gephi software was then employed to analyze these factors, determining their weights based on how frequently they were mentioned together in comments (Eq. (3).). Gephi facilitates data import, visualization, and analysis using various layout algorithms, community detection, and centrality measures to depict node connections through visual network representations.

It integrates with APIs such as the X.com API for data collection related to specific topics or hashtags [52]. Factors and their relationships were recorded in Excel and imported into Gephi to calculate network parameters like closeness centrality, betweenness centrality, hub-score, authority, and factor significance. This process involved constructing a matrix that assigned weights to cells based on the frequency of co-occurrence between factors in tweets.

(3) $$X=\frac{\left({Nf}_{x}\cap Nf_{y}\right)}{\left(Nf_{x}\cap {Nf}_{y}\right)+{Nf}_{x}+Nfy}$$

${Nf}_{x}$: The count of tweets related to factor x.

${Nf}_{y}$: The count of tweets related to factor y.

X: The value of each cell is determined by the combination of two factors' weights.

Betweenness centrality Identifies factors that act as bridges or intermediaries between different parts of the network. The formula for calculating the betweenness centrality of a node is (Eq. (4).):

(4) $$C_{B}\left(i\right)=\sum_{j\neq k\neq i}\frac{g_{jk}\left(i\right)}{g_{jk}}$$

$g_{jk}$: Denotes the total number of shortest paths from factor j to factor k.

$g_{jk}\left(i\right)$: Indicates the count of paths that traverse through factor k.

Closeness centrality (Eq. (5).) measures how close a node is to others in the network, indicating its efficiency in information dissemination. A higher value suggests centrality and better information flow. It's computed by summing the lengths of the shortest routes connecting the factor to all others in the graph.

(5) $$C_{C}\left(i\right)={\left[\sum_{j=1}^{N}d\left(i,j\right)\right]}^{-1}$$

d(i,j): the distance between node i and j.

Authority and Hub Scores are metrics used in network analysis to evaluate the importance of factors based on their connections within the network that in this research it was obtained by Gephi software calculations.

#### 3.2.4 Phase 7: prioritization of factors based on the relationship between factors (ISM) and network indicators (Gephi)

To prioritize factors, we compared network metrics from Gephi and ISM analysis. Factors with high Degree of Relative Prevalence (DRP) and authority were deemed priorities for attention and improvement. Using these parameters, a management matrix was devised to prioritize 17 factors. The matrix's horizontal axis represents DRP values from ISM, while the vertical axis denotes authority from Gephi network analysis.

• ***Special attention:*** Emphasizes long-term planning due to significant impact on other aspects.
• ***Significant attention:*** Requires focused efforts for maintaining positive consumer perception consistently.
• ***Minimal attention:*** Categorized as secondary for improvement.
• ***Regular attention:*** These factors should be continuously monitored and assessed in a timely fashion.

## 4 Result

### 4.1 Outcomes from phase 1, 2, and 3: the collection and analysis of tweets

**Fig. 3:**
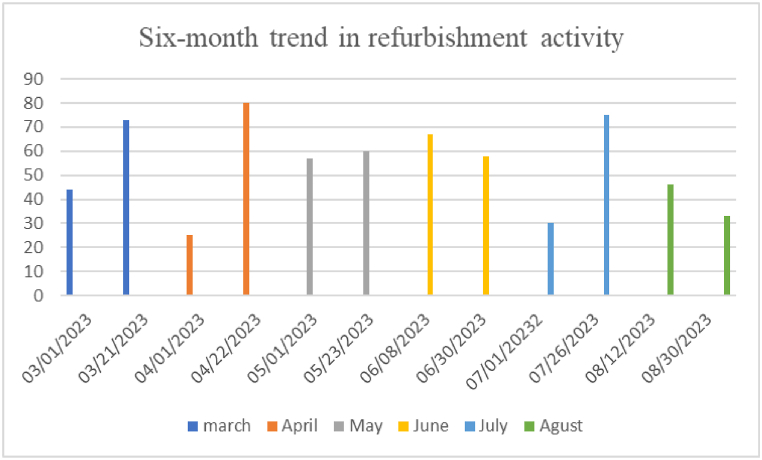
The frequency of tweets associated with the specified keywords over a six-month period.

Over a six-month period (March 2023 to August 2023), more than 65,000 tweets were gathered, encompassing user comments pertaining to the use of refurbished laptops or opinions shared on X.com regarding such products (Fig. 3).

In certain months, such as March, April, and July, the tweet rate spikes due to significant events linked to the environment like Sustainable Development, Recycling Day, World Nature Conservation Day, Earth Day, and more. The tweets in table 0.3 present challenges in analyzing relevant factors, with some being unrelated to the topic. To address this, comments were filtered using Python toolkit, resulting in refined data in Table 5.

**Table 5:** Examples of preprocessed tweets.

No.	Preprocessed tweets
**1**	not sure I will ever buy a new once again sounds like someone has joined the refurbished revolution
**2**	this was my second refurbished laptop from them I choose good in reality it was perfect
**3**	it works perfectly and delivered quickly my daughter loves it not sure I will ever buy a new one again
**4**	ahh here we go again good job acer with you worst service can't even get my refurbished laptop If this is your service refund me the amount of the laptop and keep the trash with yourself
**5**	I have only one month left refurbished laptop and display not working properly
**6**	I know there's a company here in Ireland that sell refurbished laptops called Green it for less than you'd normally pay there might be a UK equivalent
**7**	Hey it's alright I'm planning to get a refurbished apple laptop from eBay You do what you can until you can afford to get a laptop that is up to date. Apple to me is the best hence tons of 2010 on sale on eBay

**Fig. 4:**
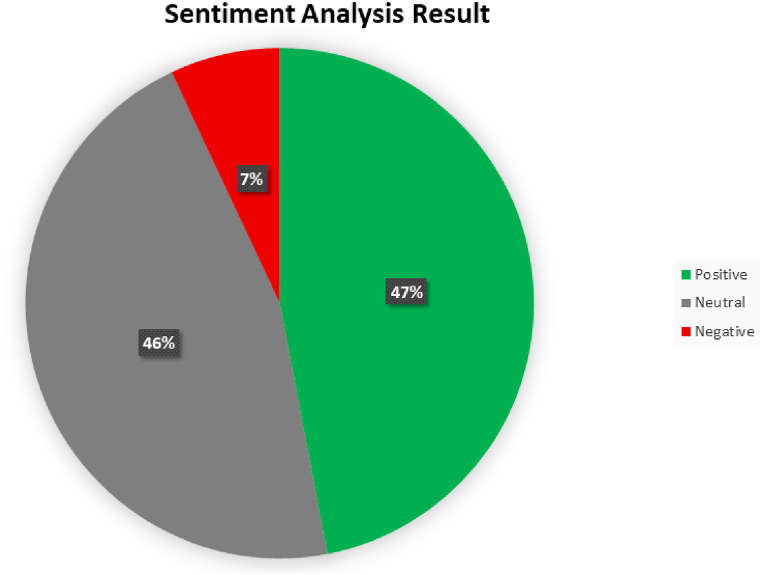
Sentiment analysis result.

The study utilized the VADER lexicon to assess emotional expression in tweets (Fig. 4). Results showed 47 % positivity, 7 % negativity, and 46 % neutrality. About 55 % of tweets received responses or retweets, suggesting agreement. Additionally, 46.1 % of tweets contained hashtags during preprocessing.

Negativity from product issues or consumer dissatisfaction may result in financial losses. A notable level of neutrality warrants scrutiny, indicating potential consumer disinterest linked to negative sentiment. Promptly addressing issues is crucial to maintain positive consumer perception.

**Fig. 5:**
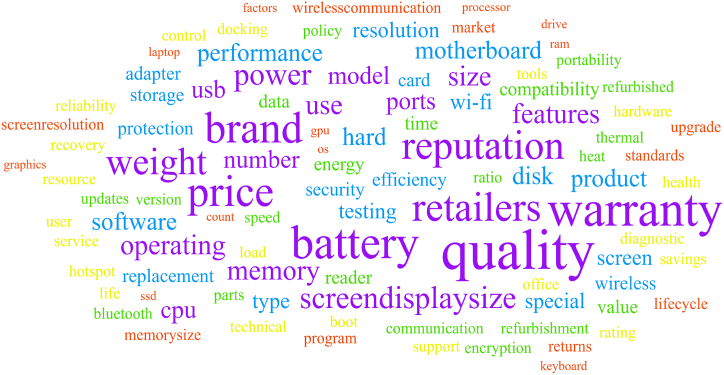
Word cloud of Keywords.

After tweet preprocessing, the factors extracted using TF-IDF were compared with previous studies, identifying seventeen key factors shaping attitudes towards refurbished products and laptops. The word cloud (Fig. 5) visually represents these key factors, highlighting their relative importance based on frequency and significance.

### 4.2 Outcomes from phase 4 and 5: relational analysis between identified factors

The relational analysis began with data collection, scrutinizing identified factors' interrelationships via Interpretive Structural Modelling (ISM). Expert consultation sought opinions on factors influencing consumer attitudes, evaluating each factor's connection. Subcategories included SSIM, reachability matrix, level segmentation, ISM-based structural model establishment, and MICMAC analysis.

Through the ISM process, relationships between factors such as price, quality, warranty, and brand reputation were mapped and categorized. This analysis was followed by MICMAC, which classified factors based on their driving power and dependence, providing further clarity on which elements of consumer perception were most influential.

#### 4.2.1 Structural self-interaction matrix (SSIM)

By harnessing the valuable insights and opinions shared by the experts (Table 6) the SSIM was formulated (Table 7).

**Table 6:** Profile of the experts.

	Gender	Age	Expertise	Year of Expertise
Expert 1	Man	38	Laptop Repairman	12
Expert 2	Man	45	Laptop Repairman	18
Expert 3	Man	30	Laptop Repairman	5
Expert 4	Man	34	Laptop seller	7
Expert 5	Man	41	Laptop seller	16
Expert 6	Man	48	Laptop seller	21
Expert 7	Man	37	University professor	5
Expert 8	Woman	45	University professor	12
Expert 9	Woman	48	University professor	13
Expert 10	Woman	42	University professor	8

**Table 7:** Structural self-interaction matrix (SSIM).

Factors	F17	F16	F15	F14	F13	F12	F11	F10	F9	F8	F7	F6	F5	F4	F3	F2	F1
**F1**	A	A	A	A	A	A	A	A	O	A	A	A	A	A	A	A	
**F2**	O	O	X	O	X	A	A	O	O	O	O	V	A	A	A		
**F3**	O	O	X	O	V	V	V	X	A	O	V	V	V	V			
**F4**	O	O	X	A	A	X	A	O	O	O	O	A	V				
**F5**	O	O	O	O	O	V	X	A	O	O	O	O					
**F6**	O	V	O	A	O	A	A	O	O	O	O						
**F7**	V	O	O	O	O	V	V	O	O	O							
**F8**	O	O	O	O	O	O	V	O	X								
**F9**	V	V	O	O	O	O	V	O									
**F10**	O	O	V	O	O	O	V										
**F11**	A	O	O	O	O	A											
**F12**	V	V	V	A	O												
**F13**	O	O	A	A													
**F14**	O	O	V														
**F15**	V	A															
**F16**	O																
**F17**																	

The experts were selected from diverse regions and professional backgrounds, including laptop repair professionals, university professors specializing in hardware and software, and retailers involved in selling refurbished laptops. These interviews were aimed at exploring factors influencing consumer attitudes toward refurbished products and offered valuable insights to complement the findings from the X.com data analysis.

The directional interrelationships between factors are represented by four symbols as follows (for factors i and j):

V: Factor i motivates the achievement of factor j.

A: Factor j motivates the achievement of factor i.

X: Factor i and j mutually motivate each other.

O: Factor i and j are unrelated.

To illustrate each symbol, consider the following examples.

1. Quality (F3) motivates the achievement of Retailers' reputation (F13) (V).
2. Price (F1) is motivated by the achievement of CPU model (F5) (A).
3. Hard disk type (F8) and Screen resolution (F17) are unrelated (O).

#### 4.2.2 Reachability matrix

The Structural Self-Interaction Matrix (SSIM) was used to create the reachability matrix, resulting in two types: the initial reachability matrix and the final reachability matrix. The initial matrix is binary, with 'zeros' indicating no interrelationships and 'ones' indicating existing interrelationships among selected factors.

The values 'V', 'A', 'X', and 'O' in the SSIM are mapped to the reachability matrix based on the following rules.

Rule 1 If the cell entry (i, j) in the SSIM is labeled as 'V', the corresponding entry (i, j) in the reachability matrix is set to 1, while the entry (j, i) is set to 0.

Rule 2 If the cell entry (i, j) in the SSIM is denoted as 'A', the entry (i, j) in the reachability matrix is set to 0, and the entry (j, i) is set to 1.

Rule 3 If the cell entry (i, j) in the SSIM is represented by 'X', the entry (i, j) in the reachability matrix is set to 1, and the entry (j, i) is also set to 1.

Rule 4 If the cell entry (i, j) in the SSIM is indicated as 'O', the entry (i, j) in the reachability matrix is set to 0, and the entry (j, i) is also set to 0.

Based on these rules, the initial reachability matrix is presented in Table 8.

**Table 8:** Initial reachability matrix.

Factors	F1	F2	F3	F4	F5	F6	F7	F8	F9	F10	F11	F12	F13	F14	F15	F16	F17
**F1**	1	0	0	0	0	0	0	0	0	0	0	0	0	0	0	0	0
**F2**	1	1	0	0	0	1	0	0	0	0	0	0	0	0	1	0	0
**F3**	1	1	1	1	1	1	1	0	0	1	1	1	1	0	1	1	0
**F4**	1	1	0	1	1	0	0	0	0	0	0	1	0	0	1	0	0
**F5**	0	1	0	0	1	0	0	0	0	0	1	1	0	0	0	0	0
**F6**	1	0	0	1	0	1	0	0	0	0	0	0	0	0	0	1	0
**F7**	1	0	0	0	0	0	1	0	0	0	1	1	0	0	0	0	1
**F8**	1	0	0	0	0	0	0	1	1	0	1	0	0	0	0	0	0
**F9**	1	0	1	0	0	0	0	1	1	0	1	0	0	0	0	1	1
**F10**	0	0	1	0	1	0	0	0	0	1	1	1	1	1	1	0	0
**F11**	1	1	0	1	1	1	0	0	0	0	1	0	0	0	0	0	0
**F12**	1	1	0	1	0	1	0	0	0	0	1	1	0	0	1	1	1
**F13**	1	1	0	1	0	0	0	0	0	0	0	0	1	0	0	0	0
**F14**	1	0	0	1	0	1	0	0	0	0	0	1	1	1	1	0	0
**F15**	1	1	1	1	0	0	0	0	0	0	0	0	1	0	1	1	0
**F16**	1	0	0	0	0	0	0	0	0	0	0	0	0	0	0	1	0
**F17**	1	0	1	0	0	0	0	0	0	0	1	0	0	0	1	0	1

To generate the final reachability matrix (Table 9), we utilized the Structural Self-Interaction Matrix (SSIM). This process resulted in two types of matrices: the initial reachability matrix and the final reachability matrix. The initial matrix is binary, with 'zeros' indicating no connections between selected factors, and 'ones' indicating existing relationships among them.

**Table 9:** Final reachability matrix.

Factors	F1	F2	F3	F4	F5	F6	F7	F8	F9	F10	F11	F12	F13	F14	F15	F16	F17	DRP
**F1**	1	0	0	0	0	0	0	0	0	0	0	0	0	0	0	0	0	1
**F2**	1	1	0	0	0	1	0	0	0	0	0	0	0	0	1	0	0	8
**F3**	1	1	1	1	1	1	1	0	0	1	1	1	1	0	1	1	0	15
**F4**	1	1	0	1	1	0	0	0	0	0	0	1	0	0	1	0	0	13
**F5**	0	1	0	0	1	0	0	0	0	0	1	1	0	0	0	0	0	11
**F6**	1	0	0	1	0	1	0	0	0	0	0	0	0	0	0	1	0	8
**F7**	1	0	0	0	0	0	1	0	0	0	1	1	0	0	0	0	1	12
**F8**	1	0	0	0	0	0	0	1	1	0	1	0	0	0	0	0	0	11
**F9**	1	0	1	0	0	0	0	1	1	0	1	0	0	0	0	1	1	16
**F10**	0	0	1	0	1	0	0	0	0	1	1	1	1	1	1	0	0	15
**F11**	1	1	0	1	1	1	0	0	0	0	1	0	0	0	0	0	0	10
**F12**	1	1	0	1	0	1	0	0	0	0	1	1	0	0	1	1	1	12
**F13**	1	1	0	1	0	0	0	0	0	0	0	0	1	0	0	0	0	13
**F14**	1	0	0	1	0	1	0	0	0	0	0	1	1	1	1	0	0	13
**F15**	1	1	1	1	0	0	0	0	0	0	0	0	1	0	1	1	0	13
**F16**	1	0	0	0	0	0	0	0	0	0	0	0	0	0	0	1	0	2
**F17**	1	0	1	0	0	0	0	0	0	0	1	0	0	0	1	0	1	14
**DNP**	17	15	11	15	14	15	7	2	2	5	11	13	12	3	14	15	10	

#### 4.2.3 Level partitions

In the next phase, the reachability matrix is segmented into levels based on three key sets: reachability, antecedent, and intersection.

A factor's reachability set includes itself and others it can potentially influence, while the antecedent set comprises factors that can influence it. The intersection set identifies factors common to both sets.

According to ISM, if a factor's reachability set matches its intersection set, it is placed at Level 1, indicating it is not influenced by factors above its level. Table A 13 in this study lists the 17 factors alongside their respective reachability, antecedent, and intersection sets.

#### 4.2.4 ISM model construction

**Fig. 6:**
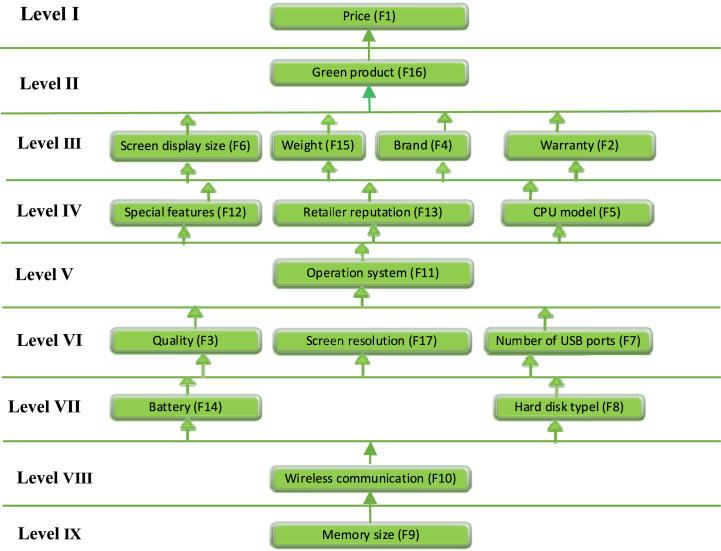
ISM based structural model for factors.

Using the final partition levels obtained from all iterations for each factor, we constructed an ISM hierarchy structure model. In the hierarchy structure model, factors (F1) occupy both the top and bottom levels. For a more comprehensive representation of this model encompassing all challenges, please refer to Fig. 6.

#### 4.2.5 Micmac analysis

**Fig. 7:**
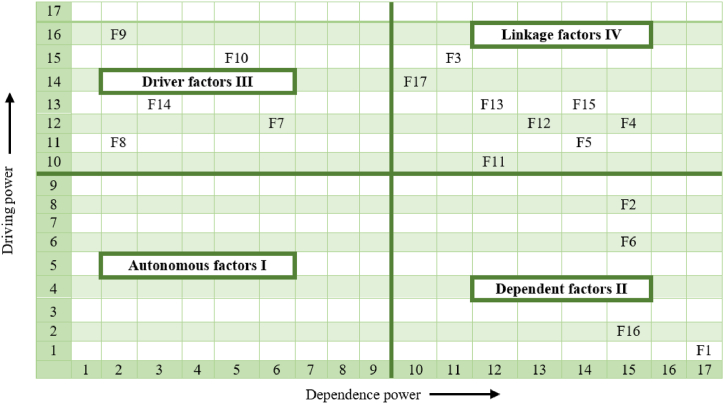
MICMAC analysis representation.

The objective of the MICMAC analysis is to generate a graphical classification of factors based on their driving and dependence power. The factors are classified into four distinct types, as follows (Fig. 7):

***Autonomous Factors***: All factors analyzed in this study were found to be interdependent, highlighting their crucial roles in shaping consumer attitudes. This suggests that each factor should be thoroughly investigated as none can be deemed less significant within this sector.

***Dependent Factors***: The factors identified as price (F1), warranty (F2), screen display size (F6), and motherboard (F16) exhibit strong dependence but limited driving power. Therefore, these factors demand greater attention and scrutiny in order to effectively address their impact.

***Linkage Factors***: Several factors, including quality (F3), brand (F4), CPU model (F5), operating system (F11), special features (F12), retailer reputation (F13), weight (F15), and screen resolution (F17), display both influential driving power and significant dependence. However, these factors also exhibit a degree of instability in their nature. Consequently, any modifications made to these factors will have a ripple effect on other factors and vice versa.

***Driver Factors***: Factors such as the number of USB ports (F7), battery (F14), wireless communication (F10), memory size (F9), and hard disk type (F8) demonstrate robust driving power but limited dependence. These factors are pivotal due to their significant influence over others, making them highly important in relation to the factors studied in this research.

### 4.3 Outcomes from phase 6: factors’ interrelationships analysis

**Fig. 8:**
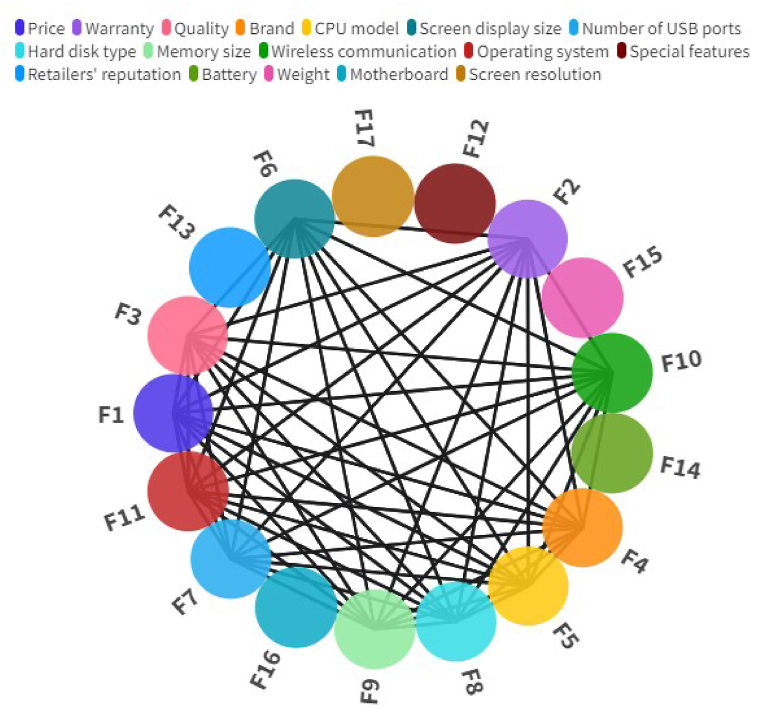
Relationships between factors.

After identifying and prioritizing factors based on mutual influence, we analyzed their communication network (Fig. 8). This analysis visualizes the relationships between factors and highlights those crucial for addressing the issue, providing insights into their complex interactions. The weights between factors (Table A 11) were calculated using Eq. (3) and imported into Gephi from an Excel file as per the methodology. The houses with values of 0 indicate that the two factors did not come together in the same tweet.

Using Eq. (4) and Eq. (5) and Gephi software, the values of Betweenness centrality, Closeness centrality, Hub-Score, and Authority were determined (Table A 12). Memory size (F9) was found to have the highest Closeness Centrality value, Screen display size (F6) had the highest Hub-Score and Authority, Warranty (F2) and special features (F12) had the highest Betweenness centrality value, Retailer reputation (F13) and Price (F1) had the lowest values of the obtained indicators among other factors.

### 4.4 Outcomes from phase 7: factors prioritization

**Fig. 9:**
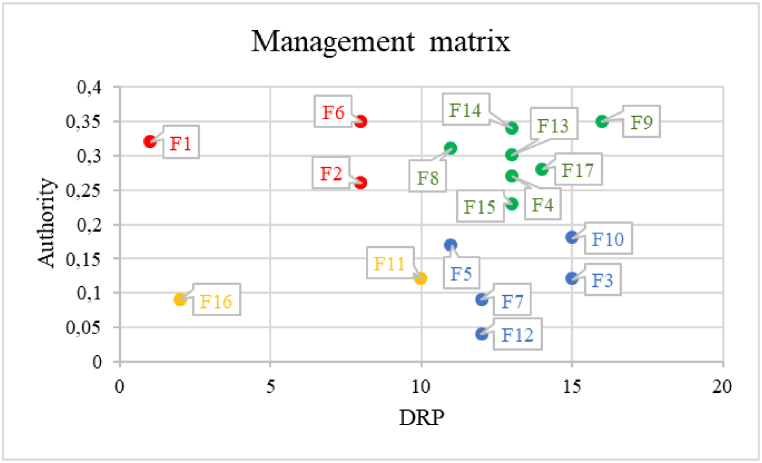
The management matrix for the final prioritization of the factors.

As a next step, we examined each factor's priority using the management matrix. To compare the results of network analysis and ISM, we only compared the value of the influence of each factor on others (DRP) with the value of the Authority, because the Authority is more reliable than other network parameters in determining the effect of a node in a graph. The vertical axis of the management matrix (Fig. 9) represents the value of DRP, and the horizontal axis represents the Authority index.

The factors are respectively, the importance and impact on consumer attitudes toward refurbished laptops, according to the management matrix:

***Special attention (Red color):*** This category encompasses Price (F1), Warranty (F2), and Screen display size (F6). Factors within this group require special attention, and long-term planning is necessary to consistently uphold consumer satisfaction.

***Significant attention (Green color):*** In this section, factors such as Hard disk type (F8), Battery (F14), Retailer reputation (F13), Brand (F4), Weight (F15), Screen resolution (F17), and Memory size (F9) exert a substantial influence on consumer decisions. Therefore, they fall into a category that demands significant attention to gain a competitive edge and reduce production costs.

***Minimal attention (Yellow color):*** Other factors like Operating system (F11) and Motherboard (F16), as revealed by the findings, are placed in this group, indicating that they are not of primary importance for improvement**.**

***Regular attention (Blue color):*** Within this group, factors like CPU model (F5), Wireless communication (F10), Special features (F12), Number of USB ports (F7), and Quality (F3) should be consistently observed and assessed in a timely manner.

### 4.5 Findings

**Fig. 10:**
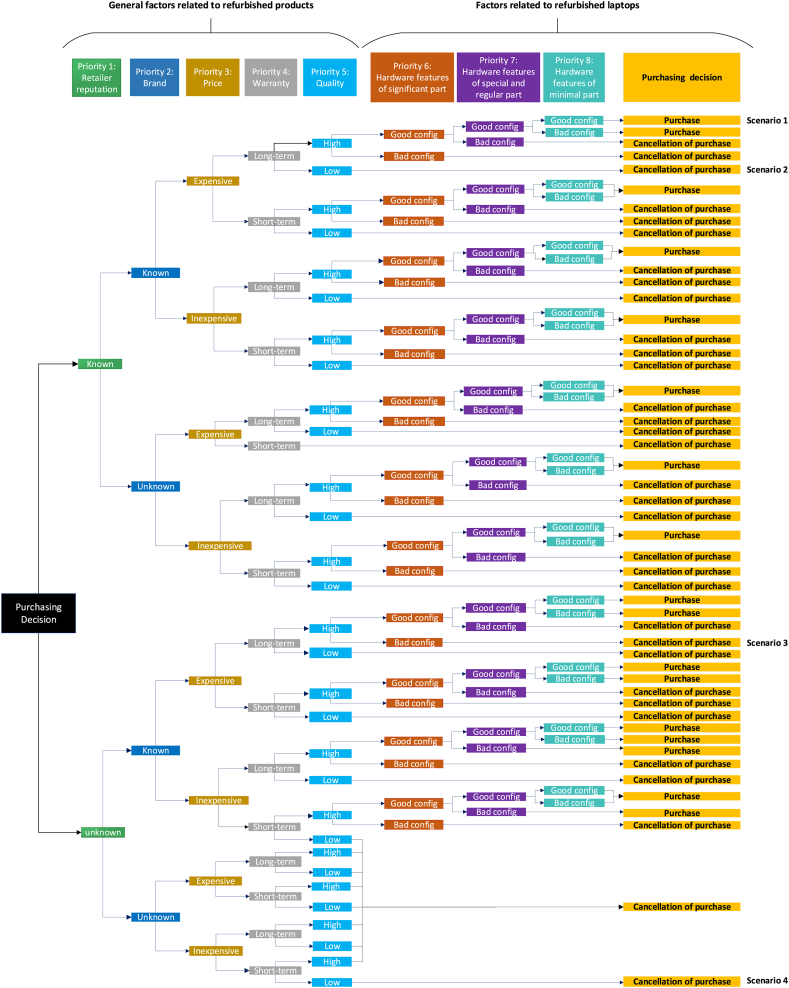
Consumers purchasing decisions.

The purchase decision model (Fig. 10) was developed by considering the opinions of experts as well as the outcomes from the management matrix. The results were derived from continuous information exchange with experts over the course of two months, which led to the categorization of the factors into two main groups: general and technological. Additionally, the factors were ranked based on their significance in the management matrix (Table 10).

**Table 10:** Categorization of factors affecting consumer decision.

General factors related to refurbished products	Factors related to refurbished laptops		
Price	Hard disk type	CPU model	Weight
Warranty	Screen display size	Memory size	Battery
Brand	Wireless communication	Operating System	
Retailer reputation	Screen resolution	Special features	
Quality	Number of USB ports	Motherboard	

This model offers manufacturers insights into the elements influencing consumer attitudes and purchase decisions. It guides producers by highlighting factors that can generate positive or negative consumer responses and identifying potential obstacles in the purchasing process. This helps manufacturers create products that satisfy consumer needs and expectations with minimal dissatisfaction. Additionally, the model underscores the significance of environmental (green product) and financial factors, which are crucial considerations for consumers [53].

This model comprises two sets of qualitative and quantitative factors, which were prioritized in the management matrix (Fig. 9). The quantitative factors encompass the hardware features of the product, while the qualitative factors pertain to the non-hardware features of the product, including retailer reputation, brand, price, warranty and the quality of the laptop. The orders of qualitative factors in the model come from the insights provided by experts in the hardware and product sales domain, which determined the priority of these factors from consumers' point of view from 1 to 5. Subsequently, the quantitative factors were prioritized as per the management matrix, with priorities assigned to numbers 6, 7, and 8.

The model prioritizes factors indicating that when consumers consider buying a refurbished laptop, their initial focus is on features that build confidence and trust. These features shape the consumer's initial attitude and encourage them to explore further aspects. For instance, if the retailer, like Amazon, is reputable, consumers are more likely to consider other factors. The model, based on a management matrix, gives equal priority to factors requiring special and regular attention due to their similar conditions. Other factors are assessed according to their positions in the matrix. To effectively use this model and understand the consumer's purchase decision process, the following explanations are provided:

When considering a refurbished laptop, the consumer first chooses a retailer, with the retailer's reputation and familiarity heavily influencing their attitude and motivation. Next, they select a brand based on their expectations, often influenced by brand authenticity and nostalgic experiences [54]. The price then becomes crucial, needing to fit within the consumer's budget. If the price is acceptable, the consumer evaluates the warranty period.

Organizations should tailor pricing strategies based on customer personalities and understand their attitudes before setting prices [55]. Warranty duration and product support can ease consumer concerns about future issues. Consumers then assess quality, evaluating the refurbished device's functionality and aesthetics [56,57]. Qualitative factors, addressing primary needs and expectations, should be optimal. Key influences on the value of refurbished products include brand, quality, price, warranty, and retailer reputation [[58], [59], [60], [61], [62]].

To illustrate the explained process, the following examples can be provided.

Scenario 1 The consumer opts to buy a branded refurbished laptop (like an Apple product), from a reputable retailer (like Amazon). The established trust in both the retailer and brand justifies the higher price. The long-term warranty further boosts confidence. The consumer then evaluates the device's quality, finding it favorable compared to other options. Once satisfied with the quality, they assess the hardware features. If these meet their requirements, the consumer proceeds with the purchase.

Scenario 2 In this scenario, the consumer considers a purchase from an unknown retailer offering no well-known brands. The high price creates a negative attitude, and a short warranty and low product quality lead them to abandon the purchase. Concerns about the quality of components from Waste Electrical and Electronic Equipment (WEEE) further deter them [63]. Instead, they choose to invest in a laptop from a reputable brand with a better warranty and higher quality.

Scenario 3 In this scenario, the consumer visits a reputable retailer for a budget-friendly refurbished laptop. Despite a long-term warranty and acceptable quality, the hardware features such as hard disk, battery, weight, screen quality, and memory capacity are unsatisfactory. Given the importance of these factors, the consumer decides against the purchase and opts to spend the same amount on a product with better hardware.

Scenario 4 Similar to Scenario 3, qualitative factors initially satisfy the consumer and encourage them to assess the quantitative factors for further consideration. However, in this case, the consumer is content with the hardware configuration in priority number 6 but finds the features in priority number 7 to be weak. The pricing of these specific factors convinces the consumer to allocate the same budget towards a product with a suitable configuration in priority number 6 and 7. Priority number 8 factors receive the least attention from the consumer and have the least impact on their attitude.

Based on the elucidated model and the potential challenges that consumers may confront during the purchasing process, manufacturers can employ related strategies to enhance each factor and enhance the overall purchasing experience.

## 5 Discussion and implications

This study offers a fresh perspective on consumer attitudes toward refurbished laptops by leveraging large-scale data analysis from social media, specifically X.com. Departing from traditional survey-based approaches, this research highlights the value of big data in capturing consumer sentiment in real time, offering a more dynamic and nuanced understanding of the factors influencing purchase decisions for refurbished electronic products. By analyzing over 60,000 tweets, we were able to identify key drivers that shape consumer attitudes, such as retailer reputation, price, and product quality. These insights, derived directly from unfiltered public opinion, present a robust and wide-reaching view of consumer priorities.

The use of X.com data provides several advantages over conventional methods, as it allows for the analysis of spontaneous, real-time consumer feedback without the constraints typically associated with structured surveys. This unstructured data captures a more authentic and diverse range of opinions, giving businesses valuable insights into consumer behavior that reflect current market trends. Previous studies [3,9] focused predominantly on questionnaires or interviews, limiting their sample size and the range of insights they could generate. In contrast, this study's approach using X.com data overcomes these limitations by accessing a much broader and more varied dataset.

While X.com data offers a wide-reaching view of consumer sentiment, the results of the expert interviews were initially considered as part of the analysis. However, upon reflection, the findings from the social media analysis proved to be more extensive and representative of real-time consumer behavior. As a result, the focus of this paper is shifted towards the X.com analysis to provide a more direct and data-driven understanding of consumer preferences. The inclusion of expert opinions, though valuable in highlighting niche aspects such as concerns over long-term quality and after-sales service, did not significantly alter the core findings drawn from social media data. Prior studies have shown that integrating social media analysis into consumer behavior research allows for capturing unfiltered and diverse opinions from a large sample size, which enhances the generalizability of the results [64]. Consequently, while the expert perspectives add context, the X.com analysis stands as the central data source driving the key findings in this study.

To address this, interpretive structural modeling and network analysis via the Gephi tool were employed to establish a design management matrix and prioritize identified factors. Moreover, to elucidate the significance of this prioritization and its implications on consumer perceptions during product purchases, a hierarchical model of consumer purchase was developed based on expert's opinions. This model illustrates how consumers simultaneously evaluate a spectrum of factors during their purchase decisions, juxtaposing their priorities against others at the point of purchase.

The study yielded three main findings: through the analysis of tweets from X.com users, factors pertaining to refurbished laptops that influence consumer attitudes and purchase decisions were extracted and categorized into “general” (retailer reputation, brand, price, warranty and quality) and “refurbished laptop” (screen display size, motherboard, CPU model, operating system, special features, weight, screen resolution, number USB ports, battery, wireless communication, memory size, and hard disk type.) factors. Consumers prioritize the following factors: retailer reputation, brand, price, warranty and quality, screen display size, motherboard, CPU model, operating system, special features, weight, screen resolution, number USB ports, battery, wireless communication, memory size, and hard disk type when purchasing refurbished laptops. Expert opinions were sought to examine the relationships and effects of the identified factors on each other. By designing a model and prioritizing influential factors, this study offers insights crucial for manufacturers to meet market demands effectively. X.com's unfiltered user comments provide a unique dataset for our analysis, complemented by expert opinions and the management priority matrix application, enhancing the depth of our contributions to the field:

Finally, the study developed a purchase decision model that emphasizes the crucial role of environmental and financial considerations for consumers when buying refurbished laptops. Our model highlights the sequential evaluation of factors, from building trust through retailer reputation and brand recognition to assessing price, warranty, and product quality.

## 6 Conclusion

This study is relevant for manufacturers that want to resell refurbished products. This research sheds light on the influential factors behind consumer attitudes towards refurbished laptops. The study aims to help manufacturers better understand consumer attitudes toward refurbished laptops to increase their purchases by identifying and prioritizing the influential factors in this process. Manufacturers must carefully consider these influential factors to enhance the refurbishment process of electronic products like laptops, align them with customer needs and preferences, and expand the market for these products. Previous research has primarily focused on the electrical and electronic equipment industry, such as mobile phones, or utilized research methodologies that were limited in their ability to gather comprehensive factors and data pertaining to consumers' attitudes towards refurbished laptops.

Through a big data analysis of X.com users' tweets, expert opinions, and the management priority matrix, the study drew several important conclusions. The analysis identified and categorized factors that have an impact on consumer attitudes and purchase decisions, distinguishing between quantitative and qualitative factors related to refurbished laptops. The study also examined the relationship and impact of these factors based on expert opinions and prioritized them using the management priority matrix. Based on the prioritization of factors, a purchase decision model was presented to guide producers in creating positive consumer attitudes and minimizing dissatisfaction and environmental impact. The model emphasized the significance of environmental and financial considerations for consumers when buying refurbished laptops.

The model highlighted that consumers initially focus on factors that build confidence and trust, such as retailer reputation and brand recognition. They then evaluate other factors, including price, warranty, and product quality. The quantitative factors, such as hardware features, were also prioritized based on their importance in the purchasing process. To enhance each factor and improve the purchasing process, manufacturers can implement various strategies. These strategies include selecting reliable retailers, establishing a strong brand identity, offering competitive pricing, providing comprehensive warranty coverage, and ensuring high product quality. Manufacturers should also prioritize hardware features based on consumer preferences and offer options that cater to diverse needs. By following these recommendations, manufacturers can positively influence consumer attitudes and purchasing decisions, ultimately leading to increased sales of refurbished laptops. This study offers useful tips for producers to improve how people view refurbished products and boost their sales. It suggests things like choosing trustworthy sellers, building a strong brand, competitive pricing, good warranties, top-notch quality, and tailoring features to what customers want. Following these suggestions can help companies shape positive consumer opinions and drive more sales of refurbished laptops.

## 7 Limitations and directions for future research

Despite the valuable insights gained from this research, there are some limitations that should be acknowledged.

First, the study primarily relied on X.com users' tweets, which may not fully represent the diversity of consumer attitudes and opinions toward refurbished laptops. X.com users may not be a representative sample of the entire consumer population, and their opinions may be influenced by various factors, including their level of engagement with technology and social media.

Second, while expert opinions were considered in the analysis, the selection of experts and their perspectives may introduce bias. The expertise and views of the selected experts could have influenced the prioritization of factors in the management priority matrix. Therefore, it is essential to recognize that different experts or a broader panel of experts may have yielded different results. Also, the results from the expert interviews should be interpreted cautiously, as the small sample size of ten experts does not allow for generalization across all consumer attitudes. These insights reflect the perspectives of a specialized group and may not capture the full diversity of consumer experiences.

Furthermore, the study focused on identifying and prioritizing factors influencing consumer attitudes and purchase decisions. It did not delve deeply into the specific strategies that manufacturers can implement to address these factors effectively. Future research could explore the implementation of these strategies in more detail, including conducting consumer surveys and market experiments to assess their impact on actual purchase behavior. To build upon the findings of this research and address its limitations, several directions for future research can be considered. Future studies can expand beyond X.com data and incorporate a more extensive range of data sources, such as online reviews, surveys, and interviews with a more diverse sample of consumers. This would provide a more comprehensive understanding of consumer attitudes and preferences.

To mitigate potential bias, future research can involve a more diverse panel of experts, representing various domains related to refurbished laptops, including environmental experts, tech industry professionals, and consumer behavior specialists. This diversity can offer a broader perspective on the factors affecting consumer attitudes. Research can delve deeper into the practical implementation of the strategies recommended in the purchase decision model. This could involve case studies of manufacturers who have successfully improved consumer perceptions and sales of refurbished laptops through the suggested strategies.

Conducting longitudinal studies to track changes in consumer attitudes and behavior over time could provide valuable insights into the evolving nature of the refurbished laptop market. This could help manufacturers adapt their strategies accordingly. Given the growing concern for environmental sustainability, future research can focus on evaluating the actual environmental impact of purchasing refurbished laptops compared to new ones. This would help consumers make more informed choices and could influence their attitudes. Expanding the research to include a global perspective would be valuable, as consumer attitudes and factors influencing purchase decisions may vary significantly in different regions and markets.

## CRediT authorship contribution statement

**Fatemeh Barkhi:** Writing – original draft, Visualization, Software, Project administration, Methodology, Data curation, Conceptualization. **Sadra Ahmadi:** Supervision, Resources, Methodology. **Sajad Shokouhyar:** Supervision, Conceptualization. **Raffaele Filieri:** Writing – review & editing, Supervision, Conceptualization. **Masoud Ramezaninia:** Validation, Supervision, Methodology.

## Additional information

Data will be made available on request.

## Declaration of competing interest

The authors declare that they have no known competing financial interests or personal relationships that could have appeared to influence the work reported in this paper.

## Figures and Tables

**Table A 11:** wt of between factors.

Factors	F1	F2	F3	F4	F5	F6	F7	F8	F9	F10	F11	F12	F13	F14	F15	F16	F17
**F1**	0	0.25	0.4	0.2	0	0.3	0	0.11	0.25	0.33	0	0	0	0.25	0.11	0	0.3
**F2**	0.25	0	0	0.27	0	0.33	0	0	0.27	0.37	0.36	0	0	0.27	0	0	0.33
**F3**	0.4	0	0	0	0	0	0	0	0	0	0.11	0.36	0	0	0.12	0	0.33
**F4**	0.2	0.27	0	0	0	0.37	0	0.14	0.3	0	0	0	0	0.3	0	0	0.37
**F5**	0	0	0	0	0	0.42	0	0.37	0.33	0	0.37	0	0	0.33	0	0	0
**F6**	0.3	0.33	0	0.37	0.42	0	0	0.37	0.33	0.5	0	0	0.4	0.33	0.33	0	0.4
**F7**	0	0	0	0	0	0	0	0	0	0	0	0	0	0	0	0	0
**F8**	0.11	0	0	0.14	0.37	0.37	0.37	0	0.27	0	0	0	0	0.27	0.3	0	0.37
**F9**	0.25	0.27	0	0.3	0.33	0.33	0.33	0.27	0	0	0	0	0.33	0.27	0.3	0	0.33
**F10**	0.33	0.37	0	0	0	0.5	0	0	0	0	0	0	0	0.37	0.17	0	0
**F11**	0	0.36	0.11	0	0.37	0	0	0	0	0	0	0	0	0.12	0	0.11	0
**F12**	0	0	0.36	0.5	0	0	0	0	0	0	0	0	0	0	0	0	0
**F13**	0	0	0	0	0	0.4	0	0.33	0	0	0	0	0	0.4	0.16	0	0
**F14**	0.25	0.27	0	0.3	0.33	0.33	0	0.27	0.27	0.37	0	0	0.4	0	0	0	0
**F15**	0.11	0	0.12	0	0	0.33	0	0.3	0.3	0.17	0	0	0.16	0	0	0.1	0
**F16**	0	0	0	0	0	0	0.11	0	0.12	0	0	0	0	0	0.1	0	0
**F17**	0.3	0.33	0.33	0.37	0	0.4	0	0.37	0.33	0	0	0	0	0.33	0	0	0

**Table A 12:** Network indicator of each factor.

ID	Hub-score	Authority	Betweenness centrality	Closeness centrality
**F1**	0.72	0.04	0.32	0.32
**F2**	0.6	0.26	0.26	0.26
**F3**	0.57	0.04	0.12	0.12
**F4**	0.6	0.08	0.27	0.27
**F5**	0.57	0.009	0.17	0.17
**F6**	0.76	0.05	0.35	0.35
**F7**	0.5	0.002	0.09	0.09
**F8**	0.72	0.06	0.31	0.31
**F9**	0.8	0.13	0.35	0.35
**F10**	0.55	0.003	0.18	0.18
**F11**	0.59	0.03	0.12	0.12
**F12**	0.45	0.002	0.04	0.04
**F13**	0.57	0.001	0.30	0.19
**F14**	0.76	0.07	0.34	0.34
**F15**	0.6	0.06	0.23	0.23
**F16**	0.55	0.02	0.09	0.09
**F17**	0.6	0.02	0.28	0.28

**Table A 13:** Level partition of reachability.

Factors	Antecedent factors set	Reachable factors set	Intersection factors set	Level
**Iteration 1**				
1	1	1,2,3,4,5,6,7,8,9,10,11,12,13,14,15,16,17	1	I
2	1,2,3,4,6,13,15,16	2,3,4,5,6,7,8,9,10,11,12,13,14,15,17	2,3,4,6,13,15	
3	1,2,3,4,5,6,7,10,11,12,13,14,15,16,17	2,3,4,7,8,9,10,12,14,15,17	2,3,4,7,10,12,14,15,17	
4	1,2,3,4,5,6,11,12,13,15,16,17	2,3,4,5,6,7,8,9,10,11,12,13,14,15,17	2,3,4,5,6,12,12,13,15,17	
5	1,2,4,5,6,11,12,13,15,16,17	3,4,5,6,7,8,9,10,11,12,13,14,15,17	4,5,6,11,12,13,15,17	
6	1,2,4,5,6,12,15,16	2,3,4,5,6,7,8,9,10,11,12,13,14,15,17	2,4,5,612,15	
7	1,2,3,4,5,6,7,11,12,15,16,17	3,7,9,10,14,15,17	3,7,15,17	
8	1,2,3,4,5,6,8,9,11,16,17	8,9	8,9	
9	1,2,3,4,5,6,7,8,9,10,11,12,13,15,16,17	8,9	8,9	
10	1,2,3,4,5,6,7,10,11,12,13,14,15,16,17	9,10	10	
11	1,2,4,5,6,11,12,13,15,16	3,4,5,7,8,9,10,11,12,15,17	4,5,11,12,15	
12	1,2,3,4,5,6,11,12,13,15,16,17	3,4,5,6,7,9,10,11,12,13,14,15,17	3,4,5,6,11,12,13,15,17	
13	1,2,4,5,6,12,13,15	2,3,4,5,9,10,11,12,13,14,15,17	2,4,5,12,13,15	
14	1,2,3,4,5,6,7,12,13,14,15,16,17	3,10,14	3,14	
15	1,2,3,4,5,6,7,10,11,12,13,15,16	2,3,4,5,6,7,9,10,11,12,13,14,15,	2,3,4,5,6,7,10,11,12,13,15	
16	1,16	2,3,4,5,6,7,8,9,10,11,12,14,15,16,17	16	
17	1,2,3,4,5,6,7,10,11,12,13,15,16,17	3,4,5,7,8,9,10,12,14,17	3,4,5,7,10,12,17	
**Iteration 2**				
2	2,3,4,6,13,15,16	2,3,4,5,6,7,8,9,10,11,12,13,14,15,17	2,3,4,6,13,15	
3	2,3,4,5,6,7,10,11,12,13,14,15,16,17	2,3,4,7,8,9,10,12,14,15,17	2,3,4,5,6,10,11,12,13,14,15,17	
4	2,3,4,5,6,11,12,13,15,16,17	2, 3, 4, 5, 6, 7, 8, 9, 10, 11, 12, 13, 14, 15, 17	2,3,4,12,15,17	
5	2,4,5,6,11,12,13,15,16,17	3, 4, 5, 6, 7, 8, 9, 10, 11, 12, 13, 14, 15, 17	2,4,5,6,11,12,13,15,17	
6	2,4,5,6,12,15,16	2, 3, 4, 5, 6, 7, 8, 9, 10, 11, 12, 13, 14, 15, 17	4,5,6,12,15	
7	2,3,4,5,6,7,11,12,15,16,17	3, 7, 9, 10, 14, 15, 17	2,3,4,5,6,7,11,12,15,17	
8	2,3,4,5,6,8,9,11,16,17	8,9	3,9,17	
9	2,3,4,5,6,7,8,9,10,11,12,13,15,16,17	8,9	8,9	
10	2,3,4,5,6,7,10,11,12,13,14,15,16,17	9,10	10	
11	2,4,5,6,11,12,13,15,16	3, 4, 5, 7, 8, 9, 10, 11, 12, 15, 17	4,5,11,12,15	
12	2,3,4,5,6,11,12,13,15,16	3, 4, 5, 6, 7, 9, 10, 11, 12, 13, 14, 15, 17	3,4,5,11,12,15	
13	2,4,5,6,12,13,15	2, 3, 4, 5, 9, 10, 11, 12, 13, 14, 15, 17	4,5,6,12,13,15	
14	2,3,4,5,6,7,12,13,14,15,16,17	3, 10, 14	3,14	
15	2,3,4,5,6,7, 10,11,12,13,15,16	2, 3, 4, 5, 6, 7, 9, 10, 11, 12, 13, 14, 15	2,3,4,5,6,7,10,11,12,13,15	
16	16	2, 3, 4, 5, 6, 7, 8, 9, 10, 11, 12, 14, 15, 16, 17	16	II
17	2, 3, 4, 5, 6, 7, 10, 11, 12, 13, 15, 16, 17	3, 4, 5, 7, 8, 9, 10, 12, 14, 17	3,4,5,7,10,12,17	
**Iteration 3**				
2	2,3,4,6,13,15	2,3, 4,5,6,7,8,9,10,11,12,13,14,15,17	2,3,4,6,13,15	III
3	2,3,4,5,6,7,10,11,12,13,14,15,17	2,3,4,7,8,9,10,12,14,15,17	2,3,4,7,10,11,12,13,14,15,17	
4	2,3,4,5,6,11,12,13,15,17	2,3,4,5,6,7,8,9,10,11,12,13,14,15,17	2,3,4,5,6,11,12,13,15,17	III
5	2,4,5,6,11,12,13,15,17	3,4,5,6,7,8,9,10,11,12,13,14,15,17	2,4,5,6,11,12,13,15,17	
6	2,4,5,6,12,15	2,3,4,5,6,7,8,9,10,11,12,13,14,15,17	4,5,6,12,15	III
7	2,3,4,5,6,7,11,12,15,17	3,7,9,10,14,15,17	2,3,4,5,6,7,11,12,15,17	
8	2,3,4,5,6,8,9,11,17	8,9	3,9,17	
9	2,3,4,5,6,7,8,9,10,11,12,13,17	8,9	8,9	
10	2,3,4,5,6,7,10,11,12,13,14,15,17	9,10	10	
11	2,4,5,6,11,12,13,15,16	3, 4, 5, 7, 8, 9, 10, 11, 12, 15, 17	4,5,11,12,15	
12	2,3,4,5,6,11,12,13,15	3, 4, 5, 6, 7, 9, 10, 11, 12, 13, 14, 15, 17	3,4,5,11,12,15	
13	2,4,5,6,12,13,15	2, 3, 4, 5, 9, 10, 11, 12, 13, 14, 15, 17	4,5,6,12,13,15	
14	2,3,4,5,6,7,12,13,14,15,17	3,10,14	3,14	
15	2,3,4,5,6,7,10,11,12,13,15	2,3,4,5,6,7,9,10,11,12,13,14,15	2,3,4,5,6,7,10,11,12,13,15	III
16	2,3,4,5,6,7,10,11,12,13,15,17	3,4,5,7,8,9,10,12,14,17	3,4,5,7,10,12,17	
**Iteration 4**				
3	3,5,7,10,11,12,13,14,17	3,7,8,9,10,11,12,13,14,17	3,7,10,11,12,13,14,17	
5	5,11,12,13,17	3,5,7,8,9,10,11,12,13,14,17	5,11,12,13,17	IV
7	3,5,7,11,12,17	3,7,9,10,14,17	3,5,7,11,12,17	
8	3,5,8,9,11,17	8,9	3,9,17	
9	3,5,7,8,9,10,11,12,13,17	8,9	8,9	
10	3,5,7,10,11,12,13,14,17	9,10	10	
11	5,11,12,13	3,5,7,8,9,10,11,12,17	5,11,12	
12	3,5,11,12,13	3,5,7,8,9,10,11,12,13,17	3,5,11,12,13	IV
13	5,12,13	3,5,7,9,10,11,12,13,14,17	5,12,13	IV
14	3,5,7,12,13,14,17	3,10,14	3,14	
17	3,5,7,10,11,12,13,17	3,5,7,8,9,10,12,14,17	3,5,7,10,12,17	
**Iteration5**				
3	3,7,10,11,14,17	3,7,8,9,10,11,14,17	3,7,10,11,14,17	
7	3,7,11,17	3,7,9,10,14,17	3,7,11,17	
8	3,8,9,11,17	8,9	3,8,9	
9	3,7,8,9,10,11,17	8,9	8,9	
10	3,7,10,11,14,17	9,10	10	
11	11	3,5,7,8,9,10,11,17	11	V
14	3,7,14,17	3,10,14	3,14	
17	3,7,10,11,17	3,7,8,9,10,14,17	3,7,10,17	
**Iteration6**				
3	3,7,10,14,17	3,7,8,9,10,14,17	3,7,10,14,17	VI
7	3,7,17	3,7,9,10,14,17	3,7,17	VI
8	3,8,9,17	8,9	8,9	
9	3,7,8,9,10,17	8,9	8,9	
10	3,7,10,14,17	9,10	10	
14	3,7,14,17	3,10,14	3,14	
17	3,7,10,17	3,7,8,9,10,14,17	3,7,10,17	VI
**Iteration 7**				
8	8,9	8,9	8,9	VII
9	8,9,10	8,9	8,9	
10	10,14	9,10	10	
14	14	10,14	14	VII
**Iteration 8**				
9	9,10	9	9	
10	10	9,10	10	8
**Iteration 9**				
9	9	9	9	9
